# Appropriateness of Pharmacologic Prophylaxis against Deep Vein Thrombosis in Medical Wards of an Ethiopian Referral Hospital

**DOI:** 10.1155/2018/8176898

**Published:** 2018-07-11

**Authors:** Mohammed Biset Ayalew, Boressa Adugna Horsa, Meseret Tilahun Zeleke

**Affiliations:** ^1^Department of Clinical Pharmacy, School of Pharmacy, College of Medicine and Health Sciences, Gondar University, Gondar, Ethiopia; ^2^Department of Pharmaceutics, School of Pharmacy, College of Medicine and Health Sciences, Gondar University, Gondar, Ethiopia

## Abstract

**Background:**

Most of hospitalized patents are at risk of developing deep vein thrombosis (DVT). The use of pharmacological prophylaxis significantly reduces the incidence of thromboembolic events in high risk patients. The aim of this study was to assess appropriateness of DVT prophylaxis in hospitalized medical patients in an Ethiopian referral hospital.

**Methods:**

Cross-sectional study design was employed. Patients with a diagnosis of DVT, taking anticoagulant therapy, and those who refused to participate were excluded from the study. Two hundred and six patients were included in the study using simple random sampling method. Modified Padua Risk Assessment Model was used to determine the risk of thromboembolism. SPSS (version 21) was used for analysis.

**Result:**

The total risk score for the study subjects ranged from 0 to 11 with a mean score of 3.41 ± 2.55. Nearly half (47.6%) of study participants had high risk to develop thromboembolism. Thrombocytopenia (platelets < 50 billion/L) or coagulopathy, active hemorrhage, and end stage liver disease (INR > 1.5) were the frequently observed absolute contraindications that potentially prevent patients from receiving thromboprophylaxis. Thromboprophylaxis use in nearly one-third (31.6%) of patients admitted in the medical ward of UoGRH was irrational. Patients who had high risk for thromboembolism are more likely to be inappropriately managed for their risk of thromboembolism and patients with thrombocytopenia or coagulopathy were more likely to be managed appropriately.

**Conclusion:**

There is underutilization of pharmacologic thromboprophylaxis in medical ward patients. Physicians working there should be aware of risk factors for DVT and indications for pharmacologic thromboprophylaxis and should adhere to guideline recommendations.

## 1. Introduction

Deep vein thrombosis (DVT) is a condition that occurs due to clotting of blood in veins located deep inside the body, mostly in the thigh and lower leg. It is also known as thromboembolism, postphlebitis syndrome, or postthrombotic syndrome [1]. DVT occurs in association with many common medical and surgical conditions. It is also the major cause of morbidity and mortality in hospitalized patients. Approximately 600,000 hospital admissions are related to DVT, and of these patients, 50,000 to 200,000 will develop pulmonary embolism [2].

Many factors are known to increase the risk of venous thromboembolism (VTE). Among these the main ones are hospitalization, hypercoagulable disorders, cancer, and surgery [1]. There are 2 types of DVT prevention methods for patients who are at risk of developing it. The first is nonpharmacologic prophylaxis like the use of compression stockings, leg elevation, sequential compression devices (SCDs), ambulation, and vena cava filter and the second is “pharmacologic,” which is through the use of blood thinning medications [1, 3]. The most common blood thinner used as DVT prophylaxis in Ethiopia is unfractionated heparin (UFH). The other well-known blood thinner, Warfarin, is not used as a primary prevention of VTE in patients who had no previous VTE; rather its use is established in the prevention of recurrent VTE in patients who already had it.

The major side effect from blood thinning medications is an increased risk of bleeding [1]. Some patients are contraindicated for blood thinning medications since they have greater risk of developing adverse events from these drugs. Depending on the risk contraindications may be either absolute or relative. An absolute contraindication exists if there is life threatening risk from use of pharmacological prophylaxis. In case of relative contraindication, caution is required while taking the drug and the benefits of therapy should be weighed against the risk in order to prescribe it. Some of the absolute contraindications for using pharmacologic VTE prophylaxis are known hypersensitivity to the drugs, current or previous heparin-induced thrombocytopenia and active bleeding, or risk of clinically significant bleeding. Relative contraindications may include (but not limited to) any risk of bleeding, creatinine clearance < 30mL/minute (reduced dosages or specific agents may be used in some cases) and use of low dose aspirin for prevention, or treatment of cardiovascular disease or other antiplatelet therapy. Nonpharmacologic prophylaxis remains an option in cases where pharmacological prophylaxis is contraindicated [4].

Most hospitalized medical patients are at particularly high risk of developing DVT and the associated complications of fatal or nonfatal pulmonary embolism and postthrombotic syndrome [5]. An increased DVT rate was reported in a significant percentage of medical patients in the absence of prophylaxis [6]. Other studies also confirm that the use of pharmacological prophylaxis significantly reduces the rate of incidence of thromboembolic events and risk of fatal and nonfatal pulmonary embolism and postthrombotic complications in medical inpatients with various other risk factors [2, 7]. Therefore, ensuring the appropriate use of prophylaxis against DVT in hospitalized patients is crucial to reduce the risk thromboembolic events and its complications [2].

Physicians in their care of hospitalized patients should appropriately assess and identify patients with risk of DVT and provide the appropriate prophylaxis in order to reduce development of DVT and its complications. Not giving prophylactic medication to a patient who deserves it will increase the risk of DVT and its complications. On the other hand if these medications are given to a patient who is not the appropriate candidate this will increase the risk of bleeding and other side effects from the drug. So initiation of pharmacological prophylaxis for DVT prevention should be done based on risk stratification. To the extent of our knowledge there is no study so far that assessed the appropriateness of DVT prophylaxis in Ethiopia. So the aim of this study was to assess appropriateness of DVT prophylaxis in hospitalized medical patients in an Ethiopian teaching hospital.

## 2. Methods

### 2.1. Study Area and Period

The study was conducted at the medical wards of University of Gondar Referral Hospital, Gondar, North West Ethiopia. Gondar is found 727 km away from the capital, Addis Ababa. University of Gondar Referral Hospital provides service in different departments like gynecology, pediatrics, dentistry, ophthalmology, psychiatry, dermatology, surgery, pharmacy, and medical laboratory. The hospital has more than 460 beds. The medical ward of the hospital is estimated to serve 1764 patients per year. The study was conducted from June 1 to August 31, 2017.

### 2.2. Study Design and Subjects

Cross-sectional study design was used to assess appropriateness of pharmacologic prophylaxis against deep vein thrombosis in medical wards of University of Gondar Referral Hospital. Patients with a diagnosis of DVT, taking anticoagulant therapy, and those who refused to participate were excluded from the study. Sample size was calculated using single population proportion formula assuming the appropriate use of thromboprophylaxis as 50 % since there is no study in Ethiopia. Correction formula was used because the source population was less than 10,000. Finally two hundred and six patients were included in the study using simple random sampling method.

### 2.3. Data Collection and Management

Data abstraction format was developed by the research authors after reviewing related literatures [8–10]. It contains participant's sociodemographic characteristics like age and sex; clinical characteristics such as diagnosis and comorbidity; and pertinent laboratory findings like platelet count and INR. Modified Padua Risk Assessment Model was used to determine the risk of thromboembolism of each patient. The risk value of 1 to 4 was given for each criteria according to its contribution for the development of thromboembolism. Total risk score was calculated by adding points given for each Padua Risk Assessment parameters seen in the patient. A total score of 4 or more indicates high risk for thromboembolism which makes the patient a candidate for pharmacologic prophylaxis provided that patient is free of any contraindication for it. List of absolute and relative contraindications taken from Venous Thromboembolism Prophylaxis Clinical Practice Guideline for Adult, Inpatient/Ambulatory, was also included in the data abstraction tool [9].

The prophylaxis was considered as inappropriate if patient was given a pharmacologic prophylaxis while he/she was not eligible or with an absolute contraindication or if he/she was not given it while it was indicated and not contraindicated for it. Data abstraction format was pretested on 5 % of the sample population after which necessary adjustments were performed before the actual period of data collection. Data was collected by two pharmacists who were trained on the basic procedures of data collection and objectives of the study. The accuracy, consistency, and completeness of the collected data were cheeked by the principal investigator on daily base.

### 2.4. Data Analysis

Data was entered in to Epi-info software (version 7) and then exported to SPSS (version 21) for analysis. Descriptive statistics was used to summarize findings and the results were presented in tables and figure. Both bivariate and multivariate binary logistic regression analysis were done to see factors associated with inappropriateness of thromboprophylaxis. All variables with p value ≤ 0.3 in bivariate analysis were taken to multivariable model to control for all possible confounders. Level of statistical significance was declared at P value ≤ 0.05 levels.

### 2.5. Ethical Consideration

The study was ethically cleared from the Ethical Review Committee of School of Pharmacy, University of Gondar. Permission letter was obtained from medical director of the hospital and presented to medical ward director. Consent form was prepared and approved by the Ethical Review Committee. As most of the information was obtained from chart and no biological sample from patients was taken consent was obtained verbally from each participant or their care giver (for patients unable to respond) after the objective of the study was communicated to them. In order to ensure the confidentiality of the information, the name and address of the participants were not recorded in the data abstraction format.

## 3. Result

### 3.1. Socio Demographic and Clinical Characteristics

From a total of 206 study subjects more than half (52.4%) were females. Majority (51.9%) were aged 18-40 years. Infectious disease was the commonest diagnosis (65.5%) while metabolic disorders were identified in 4.9% of study subjects. Description of the sociodemographic and clinical characteristics of study subjects is presented in Table 1.

### 3.2. Risk Factors for Thromboembolism

**Figure 1:**
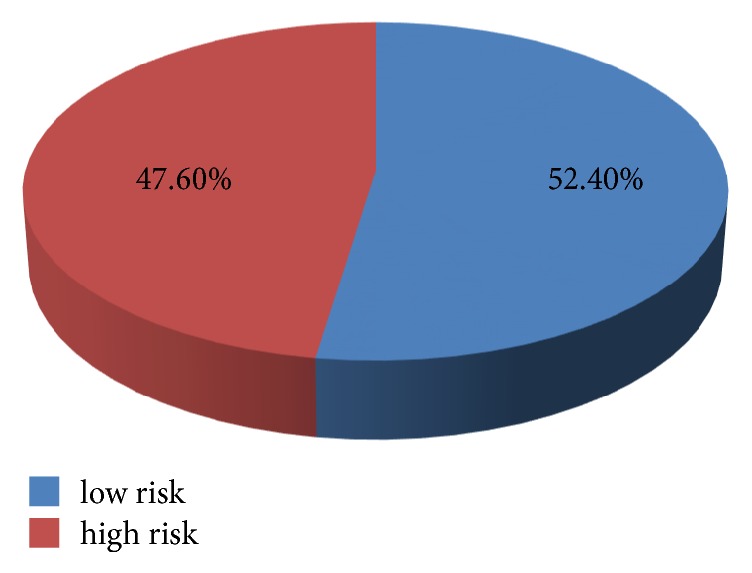
Risk of thromboembolism among study subjects.

As indicated in Table 2 acute infection (51.5%), critical illness (35.4%), heart or respiratory failure (25.7%), and reduced mobility (21.4%) were identified as the common risk factors for thromboembolism. The total risk score for the study subjects ranged from 0 to 11 with a mean score of 3.41 ± 2.55. Patients with a total risk score of 4 and above were considered as having high risk for thromboembolism. As indicated in Figure 1 nearly half (47.6%) of study participants had high risk to develop thromboembolism.

### 3.3. Contraindications for Thromboprophylaxis

Patients who had a total risk score of 4 and above should be given thromboprophylaxis unless there is a contraindication. As shown in Table 3 thrombocytopenia (platelets < 50 billion/L) or coagulopathy, active hemorrhage and end stage liver disease (INR > 1.5) were the frequently observed absolute contraindications that potentially prevent patients to receive thromboprophylaxis. Use of antiplatelets and active intracranial lesions/neoplasms was common relative contraindications in patients admitted at medical wards.

### 3.4. Appropriateness of Thromboprophylaxis

Thromboprophylaxis use in nearly one-third (31.6%) of patients admitted in the medical ward of UoGRH was irrational. As indicated in Table 4 more than a quarter (27.7%) of patients was eligible to receive thromboprophylaxis but they were not given any prophylaxis. Eight patients were given thromboprophylaxis while they are not a candidate for it.

### 3.5. Factors Associated with Inappropriateness of Thromboprophylaxis

Sociodemographic and clinical characteristics of study subjects were cheked for any association with inappropriateness of thromboprophylaxis use. As shown in Table 5 sex, age, and diagnosis were not associated with inappropriateness of thromboprophylaxis use. But patients who had high risk for thromboembolism are more likely to be inappropriately managed for their risk of thromboembolism and patients with thrombocytopenia or coagulopathy were more likely to be managed appropriately. This shows that utilization of thromboprophylaxis in high risk patients was low. Significantly lesser tendency of physicians to give thromboprophylaxis for patients with thrombocytopenia or coagulopathy is appreciated.

## 4. Discussion

The total risk score for the study subjects ranged from 0 to 11 with a mean score of 3.41 ± 2.55. Patients with a total risk score of 4 and above were considered as having high risk for thromboembolism. Nearly half (47.6%) of patients in UoGRH medical wards have risk of thromboembolism that necessitates administration of pharmacologic thromboprophylaxis. Closer to the current finding a study conducted by Khalili H. et al. reported that 38.7% of patients had significant risk of DVT so that they were candidates for pharmacologic prophylaxis [8]. Barbar S et al. also reported that 39.7% of hospitalized medical patients had high risk of VTE [11].

Acute infection, critical illness, heart or respiratory failure, and reduced mobility were identified as the common risk factors for thromboembolism. Similarly the study done by Masroujeh R reported respiratory failure, critical illness, and infection as a common risk factor for thromboembolism [10]. In contrast to this the most prevalent risk factor reported by a study done in the infectious diseases ward of Imam Referral Teaching hospital, Iran, was old age [8]. This difference may be because most (89.3%) of the patients in our study were below age of 70 years.

Patients who had a total risk score of 4 and above should be given thromboprophylaxis unless there is a contraindication. Thrombocytopenia (platelets < 50 bilion/L) or coagulopathy, active hemorrhage, and end stage liver disease (INR > 1.5) were the frequently observed absolute contraindications that potentially prevent patients from receiving thromboprophylaxis. Use of antiplatelets and active intracranial lesions/neoplasms was common relative contraindications in patients admitted at medical wards. However the study conducted at the American University of Beirut Medical Center reported that the most common relative contraindication was renal insufficiency [10].

Thromboprophylaxis use in nearly one-third (31.6%) of patients admitted in the medical ward of UoGRH was irrational. The rate of inappropriate thromboprophylaxis in our study was lower than what was reported by Amin A. et al. which reports that 72.9% of discharged patients did not receive appropriate thromboprophylaxis [12]. This variation may be because the study by Amin A. et al. assessed both pharmacologic and mechanical prophylaxis use while ours focus is on pharmacologic prophylaxis only.

Among the 29 patients who received thromboprophylaxis 8 (27.6%) had no appropriate indication. Similarly a study by Khalili H. et al. reported that 36.7 % of anticoagulants were used in the absence of an indication [8]. Drugs used as thromboprophylaxis have life threatening side effects like bleeding so they should be used only when they are indicated. By doing so we can also reduce the extra cost spent for these medications and management of their complications.

DVT prophylaxis is life saving and is also cost effective in preventing nonfatal symptomatic thromboembolism. It prevents postthrombotic syndrome which is estimated to occur in 15-40% of patients with a history of DVT [13]. DVT prophylaxis should therefore be a major consideration for hospitalized patients. Despite these potential benefits many patients are not receiving prophylaxis even though they had significant risk. More than a quarter (27.7%) of patients was eligible to receive thromboprophylaxis but they were not given any prophylaxis. According to Khalili H. et al. 18.3 % of patients enrolled in the study had indications for DVT prophylaxis but did not receive any thromboprophylaxis [8]. A study done by Ahmad H.A et al. reported that only 5% of high risk patients received appropriate prophylaxis [13].

Not giving pharmacologic thromboprophylaxis for patients who deserve it because of significant risk will impact the patient safety and will increase the occurrence of DVT which will in turn increase healthcare cost. One of the reason for not giving thromboprophylaxis in patients with high risk of DVT is forgetting to consider the risk of DVT for every patient as most of the patients had multiple diagnosis and emphasis is given for the patients' chief compliant and major diagnosis [14]. Lack of understanding of DVT risk stratification and poor knowledge of DVT prophylaxis guidelines among clinicians may also be another reason [13]. Fear of possible adverse effects from anticoagulants may also be another possible reason that may prevent clinicians from prescribing thromboprophylaxis. Since the risk of DVT occurrence in high risk patients outweighs the bleeding threat, this fear should not be a reason to avoid DVT prophylaxis [15, 16]. Pulmonary emboli may occur without any preceding attentive manifestations. So the administration of anticoagulant as prophylaxis in high risk patients should not be neglected. The result of the current study is consistent with other studies which reported that significant number of patients did not receive DVT prophylaxis while they have high risk of developing DVT [8, 12].

The result of this study indicated the need for reminding clinicians to use DVT prophylaxis guideline in preventing under- or extreme utilization of these medications and increase in overall cost. In a study conducted to assess physicians' knowledge of the management of VTE at academic medical centers, it was found that very few physicians had a sound knowledge [17]. As different studies conducted to evaluate the impact of clinical pharmacists in the rational use of DVT prophylaxis indicted a positive impact [8, 18] we recommend clinical pharmacists to be involved in the DVT risk assessment and prophylaxis recommendation in the setting.

Sociodemographic and clinical characteristics of study subjects were cheked for any association with inappropriateness of thromboprophylaxis use. Sex, age, and diagnosis were not associated with inappropriateness of thromboprophylaxis use. Similarly Barbara S reported that sex had no association with inappropriate use of VTE prophylaxis [11]. Patients who had high risk for thromboembolism are more likely to be inappropriately managed for their risk of thromboembolism and patients with thrombocytopenia or coagulopathy were more likely to be managed appropriately. This shows that utilization of thromboprophylaxis in high risk patients was low. Significantly lesser tendency of physicians in giving thromboprophylaxis for patients with thrombocytopenia or coagulopathy is appreciated.

The result of this study should be interpreted by considering the following limitation. Firstly, this study did not report on the appropriateness of prophylaxis in terms of dosage and duration of therapy. Second, the cross-sectional nature of the study limits the cause and effect relationship between independent variables and dependent variable. Lastly, it is a single center study so that the result cannot be generalized to all hospitals in Ethiopia.

## 5. Conclusion

There is underutilization of pharmacologic thromboprophylaxis in medical ward patients. Physicians working in the medical ward should be aware about risk factors for DVT and indications for thromboprophylaxis and should adhere to guideline recommendations.

## Figures and Tables

**Table 1:** Sociodemographic and clinical characteristics of study subjects.

Characteristics	Category	Frequency	Percent (%)
sex	Female	108	52.4
	Male	98	47.6

Age	18-40	107	51.9
	41-64	63	30.6
	>=65	36	17.5

Diagnosis	CNS disorder	24	11.7
	CV disease	77	37.4
	Infectious disease	135	65.5
	Metabolic disorder	10	4.9
	GI disorder	18	8.7
	Kidney disease	17	8.3
	Others *∗*	59	28.6

*∗*Cancer, asthma, COPD, schizophrenia, epilepsy, goiter, anemia, and trauma.

**Table 2:** Risk factors for thromboembolism among study subjects.

Risk factor	Points given	Patients with a risk factor (%)
Critically ill	4	73 (35.4%)
Active Cancer	3	18 (8.7%)
Previous VTE	3	1 (0.5%)
Reduced Mobility	3	44 (21.4%)
Thrombophilic Condition	3	4 (1.9%)
Recent (< 1month) Trauma/Surgery	2	4 (1.9%)
Age >= 70 years	1	22 (10.7%)
Heart or Respiratory Failure	1	53 (25.7%)
Acute Myocardial Infarction or Ischemic Stroke	1	22 (10.7%)
Acute Infection	1	106 (51.5%)

**Table 3:** Contraindications for thromboprophylaxis among study subjects.

Extent of contraindication	Contraindications	Number of patients with the condition	%
Absolute contraindication	Active haemorrhage	13	6.3
	Severe trauma to head or spinal cord, with haemorrhage in last 4 weeks	2	1.0
	Thrombocytopenia (platelets < 50 billion/L) OR coagulopathy	25	12.1
	End stage liver disease (INR > 1.5)	10	4.9
	Therapeutic anticoagulation with medication	0	0

Relative contraindication	Intracranial hemorrhage with in last year	1	0.5
	Craniotomy within 2 weeks	0	0
	Intraocular surgery within 2 weeks	0	0
	Gastrointestinal OR genitourinary haemorrhage within last month	0	0
	Active intracranial lesions/neoplasms	16	7.8
	Hypertensive emergency	3	1.5
	Post-operative bleeding concerns	0	0
	Use of antiplatelets (e.g., aspirin, clopidogrel, dipyridamole)	17	8.3
	Inherited bleeding disorder	0	0
	High falls risk	0	0

**Table 4:** Appropriateness of thromboprophylaxis use in medical wards of UoGRH.

Patient status	Frequency	Percent (%)
Eligible for thromboprophylaxis and received prophylaxis	21	10.2

Eligible for thromboprophylaxis but no prophylaxis given	57	27.7

Not eligible for thromboprophylaxis and no prophylaxis given	120	58.2

Not eligible for thromboprophylaxis but prophylaxis was given	8	3.9

**Table 5:** Factors associated with inappropriateness of thromboprophylaxis.

Variable	Category	Inappropriateness of thromboprophylaxis		COR (95% CI)	AOR (95% CI)	P value
		Appropriate	Inappropriate			
Sex	Female	69 (63.9%)	39 (36.1%)	1.565 (0.862-2.841)	1.061 (0.464- 2.430)	0.888
	Male	72 (73.5%)	26 (26.5%)	1.00	1.00	

Age	18-40	77 (72.0%)	30 (28.0%)	0.487 (0.223-1.064)	0.354 (0.071- 1.757)	0.204
	41-64	44 (69.8%)	19 (30.2%)	0.540 (0.231-1.262)	0.215 (0.042- 1.111)	0.067
	>=65	20 (55.6%)	16 (44.4%)	1.00	1.00	

CNS disorder	No	127(69.8%)	55 (30.2%)	0.606 (0.254-1.449)	0.910 (0.254- 3.259)	0.884
	Yes	14 (58.3%)	10 (41.7%)	1.00	1.00	

Infectious disease	No	43 (60.6%)	28 (39.4%)	1.725 (0.939-3.168)	1.053 (0.459- 2.415)	0.903
	Yes	98 (72.6%)	37 (27.4%)	1,00	1.00	

Critically ill	No	113(85.0%)	20 (15.0%)	0.110 (0.056-0.215)	0.766 (0.272- 2.160)	0.615
	Yes	28 (38.4%)	45 (61.6%)	1.00	1.00	

Reduced Mobility	No	117(72.2%)	45 (27.8%)	0.462 (0.232-0.916)	2.373 (0.919- 6.131)	0.074
	Yes	24 (54.5%)	20 (45.5%)	1.00	1.00	

Age>=70 years	No	127 (69.0%)	57 (31.0%)	0.785 (0.312-1.977)	6.173 (0.960-39.679)	0.055
	Yes	14 (63.6%)	8 (36.4%)	1.00	1.00	

Heart or Respiratory Failure	No	109 (71.2%)	44 (28.8%)	0.615 (0.320-1.181)	1.175 (0.468- 2.952)	0.731
	Yes	32 (60.4%)	21 (39.6%)	1.00	1.00	

Risk of the patient for thromboembolism	High	40 (40.8%)	58 (59.2%)	20.921 (8.805- 49.713)	31.67(8.518-117.72)	0.000*∗*
	Low	101 (93.5%)	7 (6.5%)	1.00	1.00	

Thrombocytopenia OR coagulopathy	No	117 (64.6%)	64 (35.4%)	13.128 (1.736- 99.307)	25.17 (2.862- 221.30)	0.004*∗*
	Yes	24 (96.0%)	1 (4.0%)	1.00	1.00	

COR: Crude Odds Ratio; AOR: Adjusted Odds Ratio; CI: Confidence Interval.

## Data Availability

The data used to support the findings of this study are available from the corresponding author upon request.

## Abbreviations

DVT: Deep vein thrombosis
INR: International Normalized Ratio
SCDs: Sequential compression devices
UFH: Unfractionated heparin
UoGRH: University of Gondar Referral Hospital
VTE: Venous thromboembolism.
